# Eliminating onchocerciasis within the Meme River Basin of Cameroon: A social-ecological approach to understanding everyday realities and health systems

**DOI:** 10.1371/journal.pntd.0009433

**Published:** 2021-06-02

**Authors:** Theobald Mue Nji, Helen Piotrowski, Nnamdi Dum-Buo, Ebua Gallus Fung, Laura Dean, Sally Theobald, Rachael Thomson, Samuel Wanji, Kim Ozano

**Affiliations:** 1 Department of Sociology and Anthropology, Faculty of Social and Management Sciences, University of Buea, Research Foundation in Tropical Diseases and Environment, Buea, Cameroon; 2 Department of International Public Health, Liverpool School of Tropical Medicine, Liverpool, United Kingdom; 3 Department of Microbiology and Parasitology, Faculty of Science, University of Buea, Research Foundation in Tropical Diseases and Environment, Buea, Cameroon; University of California, UNITED STATES

## Abstract

**Background:**

Onchocerciasis affects some of the world’s most marginalized people, perpetuating poverty and inequalities. Mass Drug Administration (MDA) with Ivermectin has taken place within the Meme River basin region in Cameroon for over 15 years. Despite this, onchocerciasis is still prevalent in the region due to existing and emerging contextual challenges. Using a social-ecological approach we explore the everyday realities of communities, highlighting the challenges and potential solutions that could support Neglected Tropical Disease (NTD) programmes when transitioning from control to elimination of onchocerciasis in this highly endemic area and other similar communities.

**Methodology/Principal finding:**

In-depth interviews (71) with community members and Community Drug Distributors (CDDs) were conducted to understand current knowledge, attitudes, and behaviours in relation to transmission, prevention and treatment of onchocerciasis. Through application of the social-ecological model, four key themes were identified: 1. Contextual factors on health promotion interventions (Onchocerciasis history and understanding of the disease, prevention and mitigation strategies and MDA experience); 2. Social determinants (poverty and livelihoods, economic and social impacts on CDD volunteers and stigma); 3. Environmental determinants (exposure, housing, occupation and poverty); and 4. health seeking pathways and decision making for treatment (access, cost and preferable treatment routes).

We discuss these core and cross cutting themes (gender differences and community participation/ownership) in relation to intersectoral collaboration, gender equity and health systems support, making recommendations for NTD programmes within the context of integrated and interdisciplinary approaches. These include the need for; intersectional and gender analysis at the local level, addressing environmental dimensions of onchocerciasis through integrated and regular health promotion, vector control strategies and access to safe water sources; reflection and action that embeds responses to social and economic barriers to MDA; integrated case detection and management that is responsive to onchocerciasis symptoms and related stigma and a fair and just support network for CDDs.

**Conclusion/Significance:**

NTD programmes need to respond to diverse community circumstances and behaviours. Communities are not a homogeneous risk group and treating them in this way will delay elimination. A deeper understanding of individual needs and their capacity to seek prevention and treatment must be considered if onchocerciasis is to be eliminated and the remaining impacts managed.

## Author summary

In Cameroon, within the Meme River Basin, ending the disease onchocerciasis, also known as river blindness, remains a health challenge, despite over 15 years of distributing the prevention and treatment drug called Ivermectin. The ongoing occurrence of the disease in this region highlights that moving from control of the disease to elimination is complicated by many factors. This research found four areas that impact on disease risk, prevention, treatment, experience and health seeking behaviour. These are 1. Contextual factors on health promotion interventions (Onchocerciasis history and understanding of the disease, prevention and mitigation strategies and MDA experience); 2. Social factors (poverty and livelihoods, economic and social impacts on CDD volunteers and stigma); 3. Environmental factors (exposure, housing, occupation and poverty) and 4. health seeking pathways and decision making for treatment. These are also affected by gender differences and community participation and ownership. We recommend the following for policy makers; intersectional and gender analysis at the local level, addressing environmental risk factors through integrated and regular health promotion, vector control strategies and access to safe water sources; action to minimise social and economic barriers to MDA; case detection and management that also addresses stigma and a fair and just support network for CDDs.

## Introduction

The Sustainable Development Goals (SDGs) recognize the elimination of Neglected Tropical Diseases (NTDs) as a target for global action. Specifically, SDG 3, ensuring healthy lives and promoting well-being for all at all ages, includes a target to end NTDs by 2030. The control and elimination of NTDs also contributes to other SGD ambitions including: poverty (SDG 1), education (SDG 4), gender equality (SDG 5), clean water and sanitation (SDG 6), decent work and economic growth (SDG 8) and inequalities (SDG 10) [1, 2]. Despite substantial progress toward combatting the global impact of NTDs, not all the targets set for 2020 and articulated within the 2012 WHO road map for the prevention and control of NTDs were met [1]. The new road map ‘Ending the neglect to attain the Sustainable Development Goals: A road map for NTDs 2021–2030’ identifies critical gaps and the actions required to reach the new 2030 targets [1]. Actions are aligned with the ambitions of the SDGs and its pledge to ‘leave no one behind,’ and identify the importance of integrating NTD activities into health systems [3, 4]. As NTDs affect the most disadvantaged and hard to reach populations, often without access to quality health services, they are considered a litmus test for universal health coverage (UHC) and an equity ‘tracer’ [1]. Onchocerciasis (or River Blindness) is an NTD that affects the world’s poorest and most marginalized people, [4, 5] deepening and perpetuating poverty and inequality by reducing the ability of individuals to earn a living and limiting productivity in the workplace [6]. Onchocerciasis is caused by the filarial parasite *Onchocerca volvulus* which is transmitted by blackflies of the genus *Simulium* which breed next to fast-flowing rivers [7, 8]

Annual Mass Drug Administration (MDA) with Ivermectin remains the key strategy in eliminating onchocerciasis in Sub-Saharan Africa [9] and was introduced in Cameroon in 1998 through Community Directed Treatment with Ivermectin (CDTI)[10]. According to the World Health Organisation, after 12–15 years, elimination of onchocerciasis is expected, provided that 80% therapeutic coverage is achieved within communities treated [11]. Elimination is defined as *‘The reduction to zero of the incidence of infection caused by a specific pathogen in a defined geographical area*, *with minimal risk of reintroduction*, *as a result of deliberate efforts*.*’ [11*, *page 9]*. The successes of MDA have been widely reported [11, 12], however, for some regions of Sub-Saharan Africa, transmission of onchocerciasis continues and alternative strategies are being considered to reach onchocerciasis elimination [13, 14]. The transmission, re-infection, diagnosis, treatment and prevention of onchocerciasis is dependent on the behaviour and livelihoods of individuals which is shaped by their physical, social, cultural, political, and economic environments [15, 16]. Greater consideration of these factors and adaptation to ongoing and emerging challenges is required; these include: civil unrest, currently ongoing in the region of this study and security concerns in west Africa more generally; hard-to-reach rural populations; co-endemicity with Loiasis as is the case in the Meme River Basin; emerging epidemics and cross-border transmission, is necessary to progress the journey toward onchocerciasis elimination [13, 17]. Thus, as many countries move towards elimination, new partnerships, collaboration and alternative approaches that respond to the realities of different contexts are required [18].

### Onchocerciasis in the Meme River Basin

The NTD Roadmap indicates that onchocerciasis in all endemic regions is targeted for elimination by 2030, however current research indicates that control approaches in Cameroon require more focused assessment and action to realise these ambitious targets[1, 19, 20]. In the Meme River Basin ongoing control and thus progress towards, elimination of onchocerciasis remains a significant public health challenge despite over 15 years of annual MDA with >65% programmatic therapeutic coverage [7, 10, 21–23]. Recent coverage data from 20 communities in the Meme River Basin show that onchocerciasis prevalence remained 44.4% with only 5.9% of participants reporting taking Ivermectin ≥ 75% of MDA rounds [7]. Additional coverage data related to the Meme Riven Basin can be found within our associated publication by Forrer et al., 2021[7]. The persistence of onchocerciasis in South-West Cameroon highlights that a transition from control to elimination of this disease using MDA is complicated by multiple factors which are well established in the literature. These include the geographical overlap with the related filarial, Loa loa. This is a risk factor for serious adverse events (SAEs) which have negative impact on community perceptions of the safety of Ivermectin (which is commonly known as ‘Mectizan’ within the study communities), and ultimately acceptance and adherence to treatment [21, 23]. Furthermore individuals experience of side effects is strongly associated with uptake of Ivermectin [24]. Other community-based factors associated with the limited impact of MDA in this area include weakening of community participation within the planning of MDA activities and poor sensitization of community members [10, 22, 24]. Potential factors limiting community participation include top-down programme management which exclude communities in the organization and planning of health activities and low community awareness of the financial management of the programme activities [25]. Health system blockages include top-down planning, poor resource mobilization, inadequate staff at frontline health facilities, funding and transparency issues and transportation challenges, all of which negatively impact on the training, supervision and monitoring of the MDA process [10, 24, 25]. These health systems bottlenecks, individual, and community factors all contribute to insufficient treatment coverage and missed treatment rounds for population groups and individuals. However, research that brings together and explores the intersections between structural, environmental, social and contextual factors and the impact these have on onchocerciasis and health systems functioning in the Meme River Basin is lacking [22, 24]. Furthermore, studies tend to use survey techniques rather than large in-depth exploration of community views analysed through an intersectional lens considering age, gender, occupation and other factors which interplay to shape experiences [2]. Kamga et al (2016) and others state that socio-anthropological studies are needed to provide additional information on the intersecting dynamics of risks to exposure and re-infection, drug availability and acceptance to understand the persistence of the disease in this area [10, 15, 16, 22, 26]. Such evidence could support health systems and other sectors to respond and adapt NTD programmes such as integrated MDA, through being responsive to diverse and changing community needs.

### Research context and approach

In this paper we review the control of onchocerciasis from different perspectives within communities, informed by the social ecological framework developed by Stokols (27) and further discussed by Gazzinelli, Correa-Oliveira (15). This framework allowed for a wider understanding of the influences on onchocerciasis experience and for making recommendations for more effective and equitable response towards managing and eliminating onchocerciasis, within and beyond the Meme River Basin in Cameroon. A social ecological lens considers the multiple levels of influence on health programmes including individual, intrapersonal, organisational, community and public policy and how these intersect with environmental aspects, social determinants, and disease specific contextual factors[27]. It is recognised that environment is a critical component of social determinants and that they are linked, however we have addressed these separately to illustrate key points. This model promotes the identification of key, often complex, issues which can lead to alternative strategies and recommendations for onchocerciasis elimination in persistently endemic communities.

**Fig 1:**
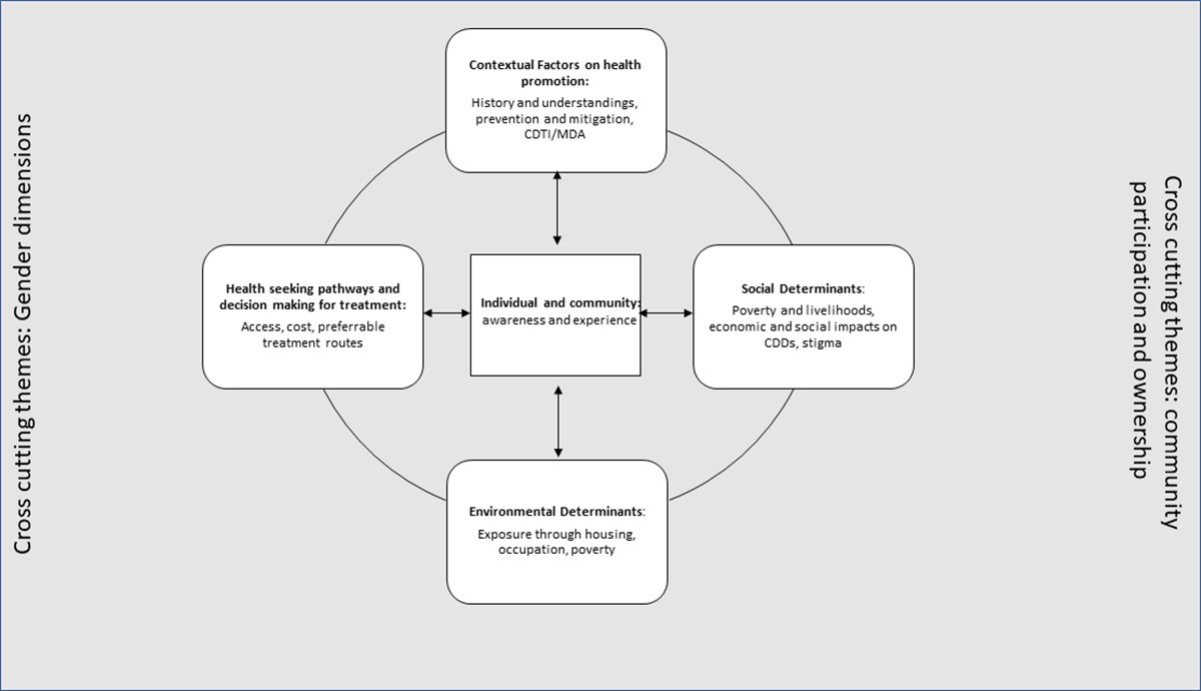
Social-ecological approach for Onchocerciasis control.

We built upon and expanded the issues outlined by Gazzineli et al [15], who proposed four interconnected thematic areas for considering effective health interventions to address NTDs These include: 1. Social determinants, 2. Environmental considerations, 3. Multi-level health promotion interventions and 4. Context specific circumstances that affect disease occurrence. Furthermore, as suggested by Gazzinelli et al [15] we examine cross cutting issues of community participation and ownership, as well as gender dimensions of health and health seeking which must be considered in the evaluation of NTD programmes, such as MDA. We chose this framework for analysis as it became apparent during data collection that individual and collective behaviours, beliefs, environmental exposures, economic circumstances, including poverty, housing location and livelihood options and social factors such as age and gender all intersected to determine how onchocerciasis risk was perceived, acted upon and controlled (or not) through medicine uptake and other means. Fig 1 highlights these concepts, which will be expanded upon in the results (Fig 1). This research was conducted as part of a wider multidisciplinary study detailed by Wanji et al [7, 21] to consider alternative strategies for onchocerciasis elimination in the region. Here we report results from the qualitative element collected during the baseline phase of the study, and apply a social ecological lens to assess the effectiveness of current onchocerciasis programmes within the Meme River Basin in Cameroon[15, 27].

We aim to highlight nuances of community practices and contextual realities that could be the difference between control and elimination in highly endemic communities such as the Meme River Basin, and make key recommendations for NTD implementers throughout the health system and their partners to consider in their aspirations to leave no one behind.

## Methods

### Ethical statement

This protocol was reviewed and approved by the Liverpool School of Tropical Medicine Research Ethics Committee (reference: 16–027), the Cameroonian National Ethics Committee for Research on Human Health (approval no. 2016/11/838/CE/CNERSH/SP), and the Division of Health Operations Research within the Cameroonian Ministry of Public Health (approval no. 631–03.17).

The objectives, importance and ethical provisions of the study were explained to the respondents with written and verbal consent obtained from literate and non-literate participants respectively before data was collected. Participants less than 18 years gave assent while their parents or guardians gave formal consent for them to participate.

### Study design

Seventy-one (71) qualitative in-depth interviews were conducted in September 2017 and then analysed to understand the daily lived realities of communities and drug distributers of Ivermectin within the Meme River Basin including their current knowledge and attitudes to onchocerciasis and their behaviours in relation to transmission, prevention, and treatment with MDA. The data collection instruments were in-depth interview guides and an observation guide. The general community entry strategies used in this study were those described by Kengne-Ouafo et al. (2014, 2015) and outlined in Wanji et al [21].

### Study site

**Fig 2:**
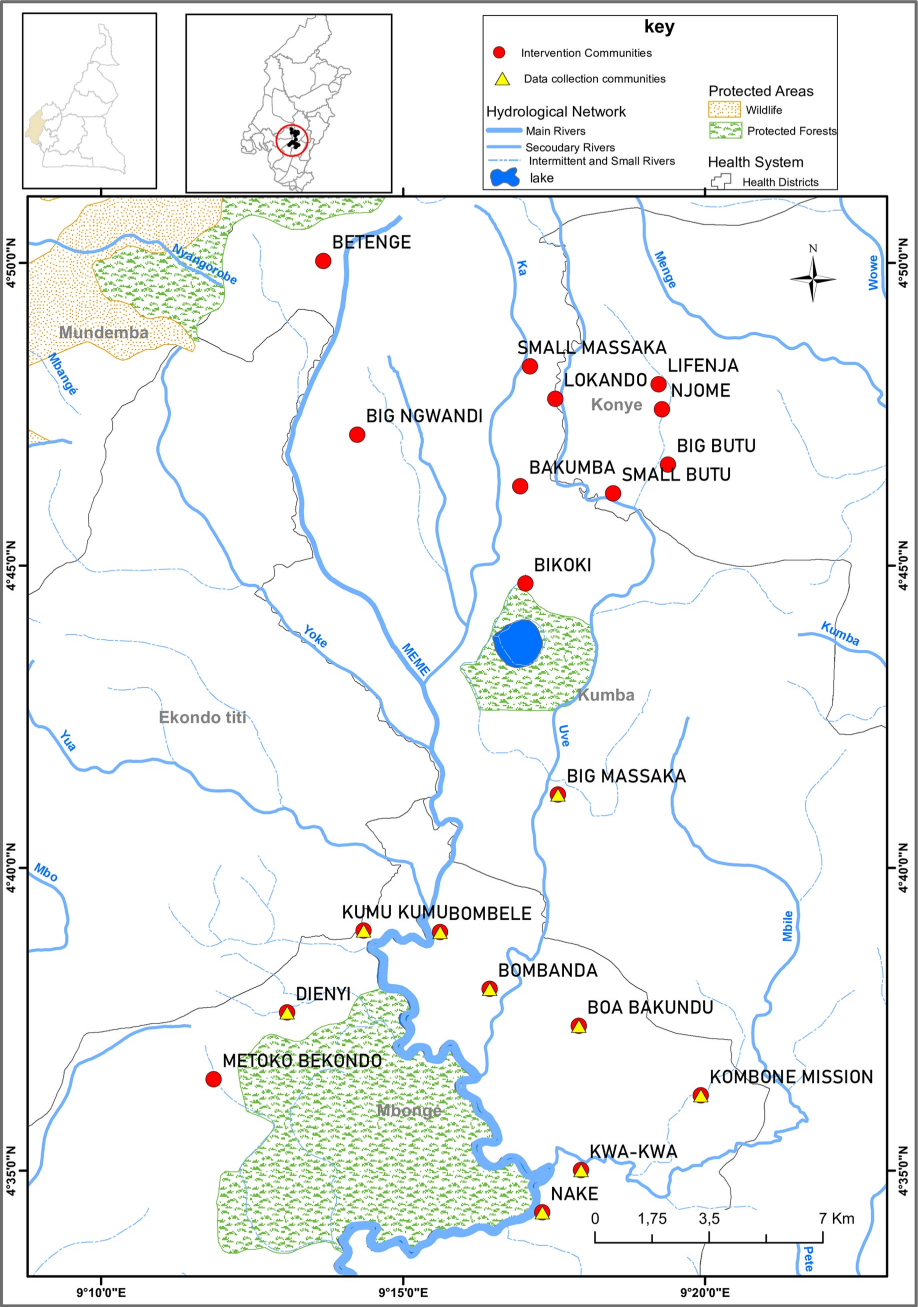
Map of the study region. Source data: Field data; Ministry of Public Health Cameroon (https://www.minsante.cm/); and Global Forest Watch (2015) under license CC BY 04 (https://data.globalforestwatch.org/datasets/f5e89ec0b0704be0a5166234aa243e92). Projection system: GCS_Clarke_1880.

This study was carried out in the Meme River Basin where there is still persistent transmission of onchocerciasis [8, 23]. The study site is located in the Rumpi Hills in the South West Region of Cameroon. The main feature is a mountain range characterised by a volcanic ridge. This ridge constitutes a watershed from which several rivers (Munaya, Meme, Mungo, Ndian, and Ube) take their waters. These fast-flowing rivers provide perennial breeding sites for the blackfly leading to continuous onchocerciasis transmission. The inhabitants of this area are in constant contact with the rivers and dense forest due to their socio-economic activities such as farming, fishing, sand mining, washing of clothes, bathing, and swimming. We purposively selected nine (9) communities from this site to be representative of the wider study (Fig 2. Source data: Field data and Global Forest Watch[28]), which included a control group where no intervention would take place, and two arms of an intervention group where different alternative strategies would be implemented[21].

### Study population

The participants selected for this study were community members and drug distributers (CDDs) residing within the Meme River Basin and who had lived for at least five years in the community (Table 1). CDDs are members of the community who are meant to be selected by the community for distribution of medications[29]. In our context, many CDDs also work on other health programmes, as detailed in our results section. Here the focus is on distribution of Ivermectin to the communities. Purposive sampling was used to select the respondents based on a sample frame guided by the principle of maximum variation (including age, gender and history of Ivermectin uptake) to ensure a wide range of perspectives were included [30]. The minimum age for all participants involved in the study was 15 years old.

**Table 1:** Summary of data.

	Community members		Community Drug Distributors	
Age (years)	Men	Women	Men	Women
15–20	3	5	0	0
21–30	4	8	4	3
31–40	4	5	8	4
41–50	3	4	1	3
51–60	2	3	2	0
60+	2	2	1	0
Sub totals	18	27	16	10
	45		26	

### Data analysis

Data was collected in both English and Pidgin. The data in Pidgin was later translated to English and together with interviews conducted in English transcribed and saved in Microsoft Word 2013 documents. Field notes and observations noted during the interviews were summarized and used to support the analysis. Transcripts were exported into NVivo 11 Pro (QSR international, 2015) and coded following a hybrid process of inductive and deductive thematic analysis to interpret raw data [31]. This analysis technique involved a process of familiarisation with the data, developing a coding framework, coding transcripts and observations to the codes, charting and synthesising the data into themes and sub-themes [30]. The data were coded into corresponding NVivo nodes (or codes) that were later exported to Excel 2013 for further analysis and synthesis into final themes presented in the results section. This approach provided a rigorous and transparent process to document the analysis [32].

## Results

Our findings support those of Gazenelli et al, highlighting that **contextual factors shaping health promotion interventions**, such as disease history and experiences within previous MDA campaigns interconnect with broader **social determinants** including stigma, poverty and economic stress to challenge the uptake and acceptability of MDA programmes and ensure adequate support for the health workforce. These challenges are exacerbated by the community’s **environmental** vulnerability and daily interaction with breeding grounds based on occupation, housing infrastructure and gendered responsibilities. Finally, barriers to health seeking such as cost and access challenges meant that **health seeking pathways and decision making for treatment** services was compromised. Our gendered analysis revealed that across all these components, gender and other factors that shape individual identity such as generation and wealth matter and influence community participation and ownership in different ways that compromise programme acceptance.

### Contextual factors on health promotion interventions

#### Onchocerciasis history and understanding of disease

The common name for onchocerciasis in our study communities is ‘filaria’, and it was described as an ongoing widespread health problem among all communities. Most participants, irrespective of age and gender, reported that filariasis is still very common in the Meme River Basin and many reported high infection rates, *“Nearly everybody in this community is infected with filaria" (*Women CDD, 21–30 years). Many community members displayed good knowledge of the symptoms of the disease including eye problems, nodules, ‘leopard skin’, rashes, epilepsy-which some evidence links with onchocerciasis[33], and body itches. Many participants also perceived that eye problems relating to filarial disease are one of the most common health problems in the area. Many reported that blindness and itching eyes are common. One community member described: “*my eyes I feel as if there are small worms inside”* (Man, community member, 41–50 years).

Mectizan is the trademarked name for Ivermectin, and is the name known in the communities. The severity of onchocerciasis, particularly skin diseases (onchodermatiatis) was reported as common in the communities as evidenced through descriptions of ‘leopard skin’. Although some community members and CDDs noted that, prior to the introduction of ‘Mectizan’ in the community, ‘filaria’ was more common and severe. There were also a few divergent beliefs that ‘filaria’ of the skin is caused by dirtiness and can be transmitted by not taking care of one’s body or by not using medicated soaps. A few women also reported that filariasis of the skin can be transmitted from one person to another via blood contact and the use of razor blades.

"Through blood and things like these razor blades. So, if you use a blade and someone else uses it, it can easily be transmitted…” (Woman, community member 41–50 years)

Some men believe that ‘filaria’ is cause by the water they use to bathe “…*If you are a dirty person*, *who does not bathe*, *you must contract filaria (Man*, *community member*, *15–20 years)*. In addition, one man reported that ‘filaria’ is a family disease or genetic disease which is hereditary.

#### Prevention and mitigation

Women reported that the fly mostly bites on the legs as women wear a gown-like dress known as the “kaba” which ends at their knees, thus exposing their legs to fly biting. Some women reported that it can be prevented by protecting oneself by wearing socks, trousers and long sleeves shirts and also by using mosquito nets.

"…Our own way is to use mosquito nets at night and when we are going to the farm we usually arm our body like to cover all part of the body so that the mosquitoes cannot bite us” (Women, community member, 30–40 years).

Some men and women saw Ivermectin as both treatment and as prevention for onchocerciasis. Whilst women CDDs reported that community members use other methods of prevention including mixing kerosene and red oil which is applied to their faces. There is also reportedly a leaf in the farm which liquid is extracted and rubbed on the face and the smell scared away the blackflies. Other prevention activities included cleaning surroundings and spraying streams and rivers with a chemical not harmful to the water organisms to kill the flies.

### Perceptions of NTD programmes

Historical factors shaped community engagement with health interventions, particularly those related to the management of filariasis. One of the women CDDs and a community member observed that even before the start of treatment with Ivermectin, people were taking a drug called “Notozine” (Diethylcarbamazine). This was considered as the drug of choice for the treatment of filariasis at the time and indicates a long history of onchocerciasis programmes within these communities. Respondents acknowledged a considerable change in their behavioural pattern towards ‘Mectizan’ through time. They outlined a number of key factors as contributing to their attrition from treatment adherence including; simple refusal to take the drug without a particular reason, absence during the distribution process, and fear of SAEs, such as death or hospitalisation.

Some participants reported that at first ‘Mectizan’ was distributed free of charge but now they are asked to voluntarily donate 100Central African Francs (XAF), which equates to 18 US cents/0.18 US dollars, before they are given the medicine. A few community members are unwilling to donate, stating that during the inception of the programme they were told that the treatment will be free of charge, and that is how they have been taking the medication over the years. This has had a knock-on effect, with many CDDs reporting that they must take the financial burden on themselves. A few also report that asking community members for money has decreased uptake of ‘Mectizan’.

Although some participants were positive and optimistic about MDA with older men reporting that it has helped in reducing the disease from the community, many women and men acknowledged that others do not like it because of the side effects which they say makes them sick:

“Like when the Mectizan are coming some people are very happy while others are so afraid that might be the sickness that is inside them which has not been disturbing them, once they took that Mectizan, it will disturb them.” (Man, CDD, 21–30 years).

Reported side effects mainly included swollen faces and legs. According to some interviewees only those with good experience of ‘Mectizan’ accept it while those that have experienced side effects, or have seen or heard others experience SAEs, will refuse to take ‘Mectizan’. This was mainly reported by young men and women, as well as farmers who fear Mectizan will prevent them completing their daily chores or will cause them harm.

### Social determinants

#### Poverty and livelihoods

Poverty and economic stressors on families necessitate livelihoods that keep them away from home for several hours and sometimes days. The communities in the Meme River Basin are typically farming communities and people spend long hours in the cocoa plantations and are unwilling to stay at home during distribution of medicines if it coincides with peak farming season. Thus, it becomes difficult to meet people at home to administer the medicine to them. Occasionally, the medicines are left for them to swallow whenever they return instead of the normal direct observed treatment (DOT) as is stipulated by the MDA process.

“Many people are negligent going there and sometimes too when you have your farm program, you will neglect going there. Door to door is most effective and if they come to your door and you are not home, they will inform you and you will have yours later.” (Man, community member, 60+years)

In addition, most communities in the Meme River Basin, especially Kumu-Kumu, have social and economic activities linked to palm wine production and/or consumption. Palm wine tapping and marketing constitute a very productive economic activity among the people in this river basin as it is a major source of income and social cohesion. Palm wine drinking huts are constructed quite close to the bushes and as they are drinking, community members can also get bitten by the blackfly (*Simulium*) the disease-causing vector.

Some community members reported that CDDs tell them that ‘Mectizan’ cannot be taken unless they do not drink alcohol for three days, and therefore a few men and women of all ages are not ready to forfeit alcohol consumption for both social and economic reasons:

“some do not respond because; one thing why some of them do not drink Mectizan is because one of the rules for drinking Mectizan is that when you drink Mectizan you should not drink ‘mimbo’ (liquor)”. (Man, CDD, 60+ years).

#### Economic and social impacts on CDD volunteers

CDDs discussed the challenges of working for a disease programme that lacked systematic financial and resource support.

“In terms of payment, we are not paid for that. It is only sometimes after distribution that the chief of post (Health centre) can call us and give us something as compensation. There is no salary.” (Man, CDD, 31–40 years)

“I find difficulty in that money does not flow for me, that is, when I want to go and get the Mectizan I stress a lot, even when I want to go and return the remaining Mectizan tablets it is also difficult. I feel reluctant at times, but I just let go because I am a first aider let me just help. The little money that I would have used to eat I use it instead to pay the transportation to and from when I want to go and take Mectizan and also when I want to return it.” (Man, CDD, 60+years).

Participants also spoke of other demotivating factors such as a lack of basic working materials like umbrella and boots during the raining season and absence of transportation logistics, materially or financially, often requiring them to spend from their already penurious pockets to have the distribution done and extend their distribution period.

The lack of financial motivation for CDDs does not only discourage those who are working but increases CDD attrition rates and discourages others who may be willing to be a distributor, thus making the workload heavy for the few willing. A woman CDD in one community explained that:

"When the chief of Centre requested six new CDDs to attend the training, it was very difficult for me to see people who could join me, one girl even told me openly that she cannot do that my type of slavery work without payment" (Woman, CDD, 21–30 years).

Also, participants revealed that some community members insult them during the distribution process making them feel alienated and presenting the work of the CDD as a pure waste of valuable time. Others associated ‘Mectizan’ distribution with the activities of the ruling party and described it as a strategic approach to support community campaigning in order to win elections.

However, both men and women CDDs were motivated to do this role to help their communities, and in support of their religious values. CDDs involved in MDA also worked in other public health programmes such as bed net distribution, polio, measles and yellow fever campaigns. Both men and women at times used their own money to support MDA including sensitisation and transport, however, demotivating factors for men included being insulted by community members.

#### Stigma

Onchocerciasis has had a very serious burden on community members, and this has also affected their behaviour and caused physical impact due to debilitating skin lesions, psychological distress due to stigma and or itching and economic impact due to reduced effective and efficient working hours and increased expenditures on health care.

The effects have been felt by men, women, and youth as they describe the manifestations of the disease and the behavioural changes that follow. Behaviour change was described as being shaped by community members’ minds and attitudes towards those with the sickness. A woman respondent supported this idea by showing the magnitude of the disease on herself, and a neighbour.

" I have seen someone who has it, I just saw how he was restless scratching his body....he does not feel free among his peers because he is always scratching his body. Some even scratch it with comb and sticks when it is worst …” (Woman, community member, 31–40 years).

### Environmental determinants

Many participants reported that the most common type of insects in the community are the *mbiti* (blackflies), also popularly referred to within the communities as ‘mosquitoes’ or red flies because of their biting nature. When prompted by researchers, community members made a distinction between the actual mosquitoes and the onchocerciasis vector, blackflies, present around them. They noted that the blackflies were the most rampant in the communities and bite them everywhere on the body and are found all over the communities, at all times of the day.

Some men noted that, ‘mosquitoes’ (local name for flies which bite) are common because of a dirty environment and swamps in and around the communities. There seemed to be a general agreement among respondents that the blackfly is mostly common around water bodies like rivers such as the Meme River and its tributaries where people usually go and bathe and carry out household chores and socio-economic activities like fishing and swimming for recreation among children and adults. But it was noted that these flies fly from these rivers into the communities where they always bite people.

"It is mostly along the riverbanks we have a big stream here called River Obe, they are so many there. That is where most people go to bathe." (Man, community member, 41–50 years)

“Mosquitoes are mostly in the night and normally follow dirt, while the black fly which gives filaria is mostly around water bodies. Like the Meme River which we have here”. (Woman, community member, 41–50 years)

Men and women had different interactions with the river, which reflected their role in family and communities. Some women reported that, they are mostly exposed to fly biting when they go to the rivers to fetch water and to wash dishes or dresses. This role is mostly associated with women and children (both boys and girls) as a household duty. Likewise, most of the communities’ lack piped water but have boreholes and wells. However, these boreholes and wells often dry up during the dry season, explaining why many people go to the river to fetch water or to wash dresses and dishes. In addition, some participants reported that they get bitten by the blackfly when they go to the river to fish as a source of income or for subsistence especially during the dry season when there are no vegetables in the farm, so they tend to depend on fishing for food.

“…There are some people in the community, mostly women and some men whose main activity is fishing they have this kind of skin… They are much exposed to where those flies are” (Man, community member, 41–50 years).

“Yes. When a lady go to wash her dresses or that of her husband, as she is concentrating to wash her dresses the mbiti will come and bite her, without her knowledge”. (Man, CDD, 31–40 years)

“I even pity but those children who are always there, at times you see so many naked children around there. Some of the little children are there from morning till evening and that’s how the mosquitoes are sucking them.” (Woman, CDD, 31–40 years)

Men reportedly get bitten when accessing the river for leisure, especially when they are having a walk around the riverbanks or when they are sitting in their compound.

“During leisure like when people sit at the veranda… when fetching water or standing or walking by the riverbank” (Man, community member, 31–40 years)

Communities in the Meme River Basin are all located in the heart of the forest with farming of cocoa and other crops as their main economic activity. Most respondents reported that their residences are generally near their farms.

‘About six days in a week only on Sundays that I do not go to the farm….The first farm is about ten minutes, and then the other one is about fifteen minutes and the one furthest I use thirty three minutes.’ (Woman, Community member 51–60 years)

Most farmers build local ovens in their farms to dry cocoa before packaging it in bags for sale. This process takes a lot of time and sometimes the farmers spend days or weeks in the farm before returning to their home or community.

“When we are breaking cocoa to remove the seed from the shell. You know at that moment we are seated in one position and the mosquitoes (blackfly) will bite a lot… “(Women, community member, 21–30 years).

Most participants agreed that blackflies concentrate in farms but are also present in their homes (particularly in villages close to rivers, such as Kumu Kumu).

### Health Seeking Pathways and decision-making for treatment

When seeking health care, there is an array of pluralistic providers to choose from including: health centre, hospital, medicine stores/community pharmacy and alternative medical or traditional practitioners and CDDs. Health seeking behaviours are hence complex, and treatment pathways are shaped by (perceived) costs and opportunity costs, trust, familiarity, embeddedness, distance, severity of symptoms and fear of operations. Despite participants in our study citing a preference for health care at the hospital or health centre when they are sick, cost of care acted as a key barrier, resulting in many choosing closer to community health care options (e.g. CDDs).

#### Preferable treatment routes

Some respondents communicated a general belief that traditional treatment (referred to by the communities as ‘country medicine’) works better than biomedical treatment for some ailments to explain why some people prefer to access herbal practices (particularly where they perceive biomedical treatment options to have failed), in addition or instead of biomedical treatment.

“I took tablets, but it did not work. So, they showed me “country medicine” [It means traditional medicine which are mostly herbs, root of plants, back of trunks etc.] which I took until I took different types of this traditional medicine, prepared them myself and drank. That is what has made it to clear like this” (Woman, Community member, 15–20 years)

Where participants did prefer to seek ‘formal care’, fear of operations or surgical treatment was reported by community members and CDDs as a deterring factor. One CDD suggested that community members would also refuse to go for nodulectomy even when it is done for free for fear that they may die.

“Some are afraid that if go through the operation, they may die” (Woman, CDD, 21–30 years).

Gendered differences were also apparent within descriptions of health seeking. For example, a few men reported that in the cases of filariasis at community level, people go to CDDs to ask for Ivermectin. "*When it occurs*, *they will rush to me and complain saying; CDD see how my skin is I suspect it is filarial*" (Man, CDD, 60+ years). Whereas a few women noted that if they diagnose a health problem and if they can handle it, they would take care of it themselves, but if they cannot they begin at the road side drug store, and if it is not resolved they proceed to the health centre. A CDD who runs a medicine store reports her experience: *“Some just come to my drug store*, *but if the issue is above me*, *I will refer them to the big Doctor [Chief of Centre] in Kombone and we will work together”* (Woman, CDD, 21–30 years). The women also recognised the extra role some CDDs play in the community by visiting people in their homes for health interventions, especially during MDA. Similarly, for side effects following acceptance of ‘Mectizan’, some community members report asking CDDs for paracetamol, or would self-administer ‘medicated soap’. Only if side effects are deemed serious would community members access health facilities.

#### Cost of services shaping a preference for community proximity

Study participants noted that long distances to health centres mean that people instead opt to buy drugs from roadside pharmacies or medicine shops locally and that choice of local provider was also influenced by pricing. For example, some women added that some people buy medicine from medicine stores because pharmacies are expensive. This view was also supported by a male CDD who acknowledged that some people practice self-medication to treat themselves because of the financial hardship they are going through. Others also use herbs (though most use herbs as enema, for hernia for example). Nonetheless, the interviewees pointed out that community-based care seeking is more common when the sickness is not serious. Men in particular, emphasised that people do not generally visit the hospital because of lack of finances even though the treatment for some of these diseases may be almost free. Generally, people are usually only taken to the hospital as cases for emergencies or when the situation becomes acute and severe.

“…they know that they have filaria but they do not do anything about it, though they scratch their bodies e.g. legs; until when it gets to the chronic stage of say poor or bad sight, they then go to the hospital” (Man, CDD, 21–30 years).

“But at times, when my child is sick and do not have enough money to go to the hospital, I take him to the pharmacy” (Woman, community member, 60+ years).

## Discussion

**Fig 3:**
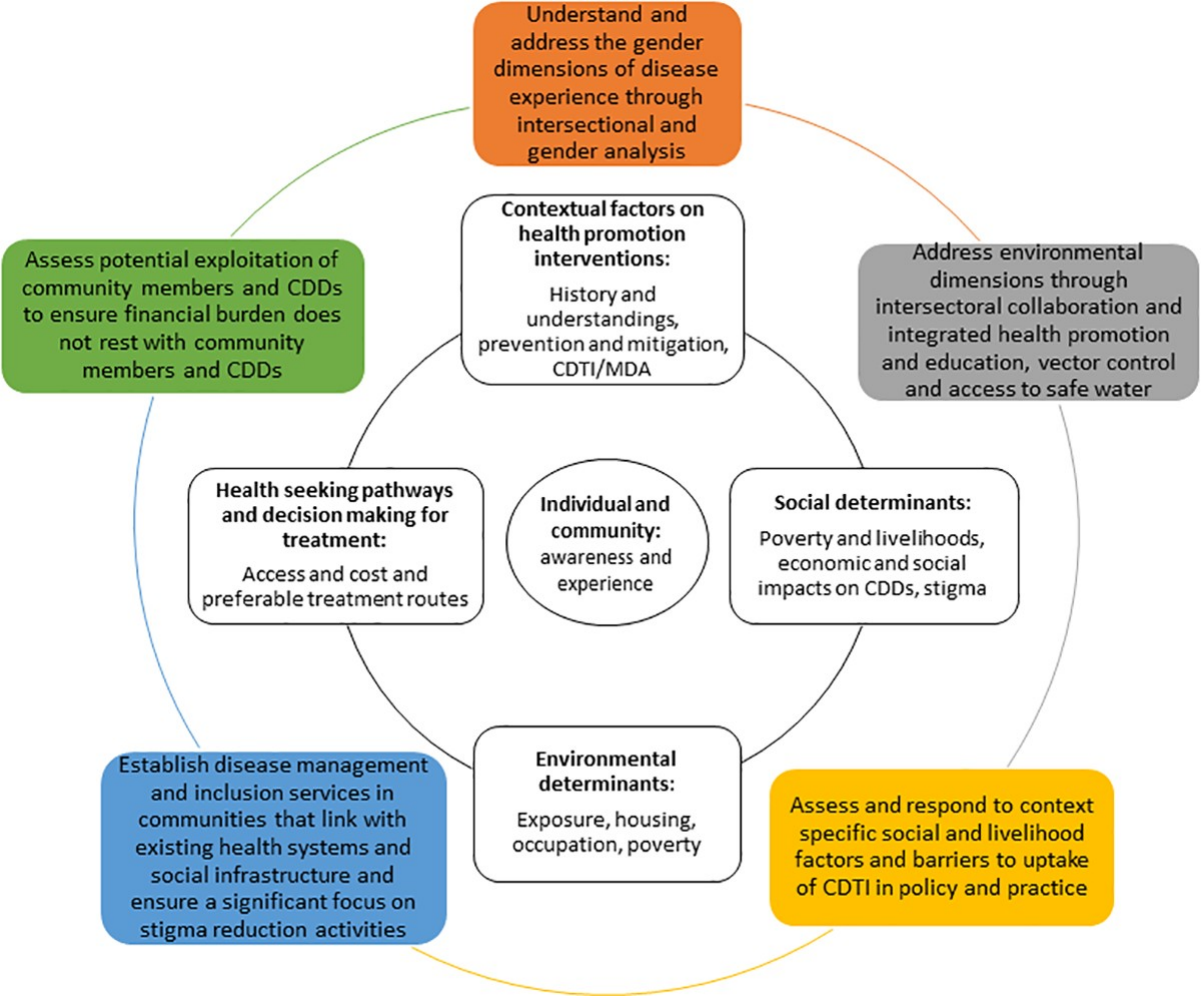
Results and Recommendations for onchocerciasis control.

The socio-ecological framework offers a lens in which to review the structural, social, environmental and contextual factors that impact on disease risk, transmission, prevention, treatment, experience and health seeking behaviour. It is clear, from evidence in the literature and confirmed here, that communities are familiar with the symptoms of onchocerciasis, and the rationale behind MDA. The communities have, over many years, observed improvements in their communities’ symptoms following MDA. However, further action at different levels that is grounded in the realities of different community members, based on their generation, gender and occupation, is required to support those affected and reach elimination. Through applying the social-ecological model and cross-cutting themes, the qualitative data highlighted several areas which could be addressed through action. Therefore, we have examined our findings alongside literature and in relation to community education, potential intersectoral collaboration, integrated service provision and health systems support, to provide recommendations and ways forward which could be considered in all health interventions for onchocerciasis. These are summarised in Table 2 and Fig 3 and discussed further below. We propose that these recommendations are useful to other highly endemic regions with similar geographical makeup and socio-economic factors, co-endemicity with loa loa, and challenges with accessing communities as well as being transferable to other MDA programmes.

**Table 2:** Recommendations and further action needed for elimination.

Recommendation for eliminating onchocerciasis	Evidence presented in this research	Further considerations for implementation
Understand and address the gender dimensions of disease experience through intersectional and gender analysis.	Women, men and children have different gendered social roles, relations and norms that impact on risk and vulnerability to, and re-infection of the disease, due to different interactions with the water, and occupational roles. Self-prevention strategies and access to treatment, also differ for men and women.	Use sex disaggregated data and frameworks to design gender sensitive programmes, consider the influence of gender specific exposure, health seeking behaviour, and with respect to who provides and receives medications.
Address environmental dimensions through intersectoral collaboration, integrated health promotion and education, vector control and access to safe water.	Women, men and children live and work close to breeding grounds for the vector increasing risk of exposure and vulnerability. Women’s, activities and household responsibilities add risk to exposure.	• Embed gender-sensitive, community-specific, health education to reduce risk and vulnerabilities. • Increase partnership with WASH and vector control initiatives. • Apply participatory methods to better understand community structures and environment which increase risk, and challenges treatment coverage, for example to limit exposure to blackflies when collecting water/washing dishes and clothes.
Assess and respond to context specific social and livelihood factors and barriers to uptake of MDA in policy and practice.	Fear of adverse events, side effects and economic consequences reduces acceptance of medications. Strategies such as house to house distribution, may reduce uptake of treatment as farmers are missed during farming seasons	• Consider including perception and management of side effect within community sensitisation and awareness raising strategies. • Engage with communities to understand how different methods of treatment distribution could be employed, including treatment support for any potential side effects. • Be less stringent with MDA timings or reporting deadlines to improve access to medications. • Consider leaving medicines in health centres to allow for longer term ‘mop up’ strategies
Establish disease management, disability and inclusion services in communities that link with existing health system and social infrastructure and ensure a significant focus on stigma reduction activities.	Economic determinants affect sources of health accessed in community. Access to health facilities is challenging. Reliance on local health providers (formal and informal) are more likely. People living with symptoms of onchocerciasis experience distress and stigma.	• Consider who and where health services and treatment are best placed to provide disease management and wellbeing support within the communities. • Work with local providers to promote appropriate health seeking. • Ensure that stigma reduction activities are implemented alongside MDA to support the inclusion of people affected by chronic disease and disability in MDA. • Improve access to disease management, disability and inclusion services by creating stronger links between communities and the health system as well as supporting the training and capacity strengthening of CDDs and other formal and informal providers to provide such services at community level.
Assess potential exploitation of community members and CDDs to ensure the financial burden does not rest with community members and CDDs	CDDs out of pocket expenditure leads to attrition of workforce, increased poverty for workforce and change in respect and relationship with community members which will impact on medicines uptake	• Work with funders and donors to address the funding gaps. • Explore and embed adequate enablers and incentives to the workforce

### Recommendation 1: Understand and address the gender dimensions of disease experience through intersectional and gender analysis at a local level

Throughout the results and in the literature, we see that gender roles and relations shape all aspects of onchocerciasis elimination [34, 35]. The distribution of social roles by gender is highlighted here and demonstrated in terms of how differential risks are present for women and men, boys and girls as they interact with their environment in different ways for work, play and household tasks [34, 36]. Gender, sex and their intersections with other social determinants of health shape peoples’ vulnerability to and experience of NTDs, as well as their ability to access care and treatment [2, 37]. Gender and intersectional analysis which aims to understand how the dimensions of age, gender, poverty and other factors intersect with environment to impact transmission, re-infection and access to prevention and treatment is needed to better equip and accelerate localised responses to deliver equitable prevention, diagnosis, and treatment services in the Meme River basin [38]. This could be facilitated by applying gender analysis tools and frameworks [2, 39]. The importance of collecting and reporting sex disaggregated data, and utilising this to strengthen NTD programmes has previously been highlighted by Theobald et al., including gendered considerations for the workforce [2]. In our study both women and men CDDs expressed their motivators and demotivators for continuing with their role. Local level analysis should consider what strategies could better support CDDs and consider gender dimensions which may impact on a sustainable workforce.

### Recommendation 2: Address environmental dimensions through intersectoral collaboration, integrated health promotion and education, vector control and access to safe water

The communities that live near to the rivers experience frequent fly biting everywhere and all the time; during economic, social and leisure activities. Adults in these communities are reliant on the rivers for everyday living, as well as being severely limited in their ability to avoid the water sources due to the close proximity of their farms and homes to the rivers. School aged children and young adults often spent long hours without protective clothing by the river increasing their risk. There are potential actions and solutions that could address some of the environmental conditions found here as bottlenecks to eliminating onchocerciasis. These include gender sensitive, frequent and ongoing health education to reduce risks and vulnerabilities, vector control strategies, intersectoral collaboration with sectors such as Water, Hygiene and Sanitation (WaSH), housing, planning and others and increasing access to safe water sources [40] which we explore further below.

### Gender sensitive, frequent and ongoing health education to reduce risks and vulnerabilities

Health education, when integrated with other sectors/disciplines such as WaSH, planning, housing and vector control activities can be key to sustaining the benefits of medicines, and preventing infection [15]. Working alongside local organisations, religious institutions and other trusted community structures can improve access and uptake of information. Participatory methods to understand how different community groups interact with the structures around them would support these efforts, as done in Nigeria using transect walks and social mapping techniques [41]. For example, understanding working patterns and reaching specific groups of workers at their locations would be beneficial. Targeted and gender sensitive materials and messages for women, men and children could be researched and co-designed in collaboration with CDDs alongside a review of information, education and communication materials [41, 42].

### Vector control

Vector control strategies are recommended in the bordering districts of the Centre and West Regions of Cameroon, as well as in other parts of the country with persistent high prevalence in order to reach onchocerciasis elimination [20]. Walker et al. [43] stress the importance of vector control in high-transmission settings as a complementary intervention strategy to MDA, especially where suboptimal responses to ivermectin are found. The Global Vector Control Response 2017–2030 proposes that for vector control strategies to be successful they need to be embedded in; strong inter and intra sectoral collaboration and action; engage and mobilise communities; have robust surveillance, monitoring and evaluation interventions; and use scalable and integrated tools and approaches[44].

### Access to safe water sources

Whilst wells and boreholes were present in these communities, they were often dried out, meaning that community members (especially women) access the rivers where blackflies are breeding for everyday living. Whilst integration and collaboration of WaSH and NTD actors has been advocated for many NTDs such as Trachoma, Schistosomiasis, Lymphatic Filariasis and Soil Transmitted Helminths [45–48] this research suggests that further exploration is needed for how WaSH integrated strategies may reduce risk and exposure to onchocerciasis, particularly in areas which remain endemic despite more than 15 years of MDA. Furthermore, in these communities, visual impairment and blindness (whilst reduced in prevalence) remains a concern. Therefore, accessible safe water and sanitation for these communities is also needed [48].

### Recommendation 3: Assess and respond to context specific social and livelihood factors and barriers to uptake of MDA in policy and practice

In accordance with other studies, farmers expressed caution to accepting medication due to fear of SAEs and side effects which will have economic consequences in being unable to work or needing further treatment[7, 16]. In these regions, historical memory of SAE potentially linked with co-endemicity of Loa loa, may heighten fear of associated reactions, and lead to reduced uptake of MDA [7, 23, 49]. Adequate support and management strategies for potential or perceived side effects should be considered [7].

Furthermore, communities migrated for work meaning they were often absent during distribution times, and social barriers to accepting Ivermectin such as alcohol use and related sales will all have an impact on elimination goals. Small changes that respond to context specific social and economic barriers may be the difference between success and failure of elimination efforts. Suggested adaptations to NTD programmes that could be negotiated with donor and implementing partners, to tackle some of the challenges related to these social determinants include being less stringent about MDA timings or further flexibility in the reporting deadlines; in campaign structure so that specific populations who are currently missed can be reached outside of campaign periods, through for example, some medicines being left in health centres to allow for longer term ‘mop up’ or repeat visits strategies[16].

### Recommendation 4: Establish disease management, disability and inclusion services in communities that link with existing health system and social infrastructure and ensure a significant focus on stigma reduction activities

Although this research aimed to understand bottlenecks for MDA, the data revealed that many community members are still living with severe onchocerciasis symptoms, including stigmatising skin conditions and eye problems. This highlights that disease management, disability and inclusion strategies are also needed as well as preventative strategies. Prevention strategies should also prioritise the inclusion of people experiencing morbidity and disability. However, in considering disease management, disability and inclusion strategies, it is important to understand how and when different community members access health care, the values placed on different health providers (and why), and the implications this has on a specific disease within a given context. Understanding how relational gender roles intersect with other social factors and wider determinants of health, within specific contexts, and how they may influence health seeking pathways is important [35, 39, 50], and should be considered in all NTD programmes.

This research exposed the range of formal and informal health providers that communities accessed, and the influences on decision making when seeking care. These were related to trust, convenience, cost, travel limitations and balancing the benefits of treatment against reductions in the ability to earn a living. These insights have important implications for all health programmes, including MDA, disease management, disability and inclusion and health promotion strategies.

Furthermore, delays in seeking health care were due to inaccessibility of health facilities, but also fear of associated economic and personal costs of seeking care. Within this research, community members expressed the stigmatising effects for people living with onchocerciasis in the Meme River Basin. Research on NTDs and stigma in Liberia has demonstrated that for people living with NTD morbidity and disability, it was socially acceptable to have initial sickness, however as their illness became more permanent, people described significant negative impacts on their mental-wellbeing, including depression, anxiety and suicide [51]. This delay in health seeking behaviour until symptoms are severe, coupled with limitations in MDA uptake means that preventative strategies alone are not sufficient, and that case detection and disease management strategies are required to adequately complement preventive chemotherapy interventions [43, 51, 52]. Inclusion of people with disability within prevention campaigns is also essential in reaching elimination targets.

### Recommendation 5: Assess potential exploitation of community members and CDDs to ensure financial burden does not rest with community members or CDDs

Farming communities are sometimes inaccessible, especially during the wet season, and some respondents reported that they would stay for long periods on their farms. This limits who can be reached by traditional house to house distribution methods and highlights the need to employ new methods of medicine distribution which are context specific, such as fixed-point distribution [16, 53]. CDDs and health facilities could be left with medicines for those who return from work, however current donor restrictions limit this, which places additional burden on CDDs [16]. CDDs still lack sufficient resources to do their roles, such as umbrellas and boots which are needed to reach some communities in order to increase coverage, nevertheless even with appropriate resources this involves long treks, which impacts of on CDDs’ time and other economic activities. Furthermore, the functioning of CDDs as volunteers, who live and work in some of the poorest communities in Cameroon, risks exploitation and potential exacerbation of social and gender inequities.

The financial and resource challenges faced by CDDs in MDA programmes identified here confirms a well-established evidence base beyond Cameroon, including out of pocket spending, transport restrictions and resulting physical and mental stresses from working in challenging environments when delivering medicines [54, 55]. Yet these reported challenges persist. CDDs play a critical role in NTD programmes, but relying on volunteerism, without adequate enablers and incentives is not a sustainable solution, and the international community have a responsibility to promote equitable solutions to the control and elimination of NTDs and to ensure that community members who want to support their communities to deliver medicines are not exploited.

As seen here, some CDDs are sacrificing food to deliver medicines and refer to the work as *a ‘type of slavery work without payment’*. This clashes with SDGs and should not be ignored as we move forward to achieve UHC. Whilst community ownership is paramount to the success of MDA [56, 57] this does not mean that the financial burden should rest with community members and CDDs, and highlights the need for well-funded, sustainable programmes that respect the wellbeing of volunteers. Duamor et al (2017) highlights that renumeration for CDDs was initially provided through the government and a shift in responsibility to communities to compensate this cadre occurred in 2013, however communities were not aware that government payments had ceased and were reluctant to support CDDs, possibly suspecting CDDs are asking for double remuneration[10]. Our results and published literature show that under funding for the NTD programme is a long-standing issue which needs urgently addressing and actions put in place to address this issue. Furthermore, a recent review, including evidence from Cameroon, emphasised that despite a reliance on CDDs to implement national NTD strategy, there is limited guidance or delineation of what this should look like in practice, such as with motivational support for CDDs. The lack of policy and procedure in this regard impairs the effectiveness of national programme strategy[29].

### Limitations

We experienced some challenges during data collection, including being unable to reach one community (Metoko Bekondo) due to inaccessible roads. This highlights the importance of consideration of environmental factors, which can have a significant impact on geographical coverage for elimination strategies. Interviews were not conducted with community members who are ineligible for MDA with Ivermectin (such as pregnant women), due to limitations in recruitment strategies. Inclusion of these individual perspectives would strengthen alternative strategies to reach ineligible populations and should be considered in subsequent research. In addition, interviews were not conducted with health systems actors at this stage. Future research should engage Ministries of Health and other health systems stakeholders to communicate the programmatic challenges found here and engage them in joint solution development as discussed elsewhere [42, 58]. Cost analysis was not conducted as part of this study; however, we recognise that this should be considered in any further research that seeks to implement and evaluate our recommendations.

## Conclusion

In conclusion, there are many reported nuances of understanding and behaviours shaped by gender, age and occupation which are still not being actively addressed by localised NTD programmes. Understanding community risk as homogeneous will not lead to elimination, and therefore a deeper understanding of individual needs and their capacity to seek prevention and treatment must be considered if onchocerciasis is to be eliminated.
